# From human writing to artificial intelligence generated text: examining the prospects and potential threats of ChatGPT in academic writing

**DOI:** 10.5114/biolsport.2023.125623

**Published:** 2023-03-15

**Authors:** Ismail Dergaa, Karim Chamari, Piotr Zmijewski, Helmi Ben Saad

**Affiliations:** 1 Primary Health Care Corporation (PHCC), Doha, Qatar; 2 Research Unit Physical Activity, Sport, and Health, UR18JS01, National Observatory of Sport, Tunis 1003, Tunisia; 3 High Institute of Sport and Physical Education, University of Sfax, Sfax, Tunisia; 4 Aspetar, Orthopaedic and Sports Medicine Hospital, FIFA Medical Centre of Excellence, Doha, Qatar; 5 Jozef Pilsudski University of Physical Education in Warsaw, Warsaw, Poland; 6 University of Sousse, Farhat HACHED hospital, Service of Physiology and Functional Explorations, Sousse, Tunisia; 7 University of Sousse, Farhat HACHED hospital, Research Laboratory LR12SP09 «Heart Failure», Sousse, Tunisia; 8 University of Sousse, Faculty of Medicine of Sousse, Laboratory of Physiology, Sousse, Tunisia

**Keywords:** Artificial Intelligence, Chatbot, Deep Learning, Higher Education, Google Bard, LLaMA, LLM, Machine Learning, Natural Language Processing, NLM, NLP, Paperpal, Peer Review, QuillBot, Rayyan, Research, Sports Medicine

## Abstract

Natural language processing (NLP) has been studied in computing for decades. Recent technological advancements have led to the development of sophisticated artificial intelligence (AI) models, such as Chat Generative Pre-trained Transformer (ChatGPT). These models can perform a range of language tasks and generate human-like responses, which offers exciting prospects for academic efficiency. This manuscript aims at (i) exploring the potential benefits and threats of ChatGPT and other NLP technologies in academic writing and research publications; (ii) highlights the ethical considerations involved in using these tools, and (iii) consider the impact they may have on the authenticity and credibility of academic work. This study involved a literature review of relevant scholarly articles published in peer-reviewed journals indexed in Scopus as quartile 1. The search used keywords such as “ChatGPT,” “AI-generated text,” “academic writing,” and “natural language processing.” The analysis was carried out using a quasi-qualitative approach, which involved reading and critically evaluating the sources and identifying relevant data to support the research questions. The study found that ChatGPT and other NLP technologies have the potential to enhance academic writing and research efficiency. However, their use also raises concerns about the impact on the authenticity and credibility of academic work. The study highlights the need for comprehensive discussions on the potential use, threats, and limitations of these tools, emphasizing the importance of ethical and academic principles, with human intelligence and critical thinking at the forefront of the research process. This study highlights the need for comprehensive debates and ethical considerations involved in their use. The study also recommends that academics exercise caution when using these tools and ensure transparency in their use, emphasizing the importance of human intelligence and critical thinking in academic work.

## INTRODUCTION

In the last decade or so, the field of artificial intelligence (AI) has seen remarkable advances, and Chat Generative Pre-trained Transformer (ChatGPT – open AIs GPT-3 model) is a striking example of this progress [1]. Academic research has traditionally relied on laborious manual methods to sort and analyse large volumes of text. However, recent advances in natural language processing (NLP) technology have made it possible to automate many of these tasks. ChatGPT is one such technology that has shown promising prospects in academic research. ChatGPT is a large language model (LLM) that has been trained on an extensive corpus of text, enabling it to generate human-like text responses. For a few years now, it has been evident that AI can produce coherent language, and it is becoming increasingly challenging to distinguish AI sentences from those created by humans. In 2022, the journal *Nature* reported that scientists were already using chatbots as research assistants to help them organize their thoughts, receive feedback on their work, write codes, and even summarize research literature [2].

ChatGPT has the ability to create well-written student essays, summarize research papers, answer questions well enough to pass medical exams, and generate helpful computer codes, for instance [2]. It has even created research abstracts that scientists found difficult to distinguish from those written by a human [2]. However, this technology has also the potential to produce spam, ransomware, and other harmful outputs, which is substantially worrisome for our societies [2]. Given the potential for LLMs, like ChatGPT, to disrupt various fields, there is an urgent need for the research community to engage in a comprehensive debate on the potential uses, threats and limitations of these tools. Therefore, the aims of this correspondence were to *(i)* provide an overview of ChatGPT and other NLP technologies, their emergence, limitations, and related practical implications; and *(ii)* examine the prospects and consequences of using these tools in academic writing

## MATERIALS AND METHODS

This manuscript was based on a literature review of relevant scholarly articles, only published in peer-reviewed journals indexed in Scopus as quartile 1. The search was conducted using keywords such as “ChatGPT,” “AI-generated text,” “academic writing,” and “natural language processing”. To ensure the reliability and quality of the sources used in this paper, we excluded preprints from our references. Indeed, the lack of pertinence of the latter and the potential bias they may contain, particularly with the recent emergence of National Library of Medicine (NLM) technologies did not ensure that they would provide a trustworthy and unbiased assessment of the state of the field. The analysis was carried out using a quasiqualitative approach, which involved reading and critically evaluating the sources, and identifying relevant data to support the research questions.

## RESULTS AND DISCUSSION

### Is NLP technology, including ChatGPT, really a new concept?

NLP has been an area of study since the dawn of computing. The creation of electronic computers in the mid-20^th^ century marked the first attempts to develop computer programs capable of comprehending and generating human language. The roots of products like ChatGPT lie in early AI research from the 1950s and 1960s [3]. Scientists have been aiming at creating computer programs that could understand and respond to natural language in a manner that mimics human communication. This task was challenging due to the complexity, ambiguity, and variability of human language with complex cultural influences that rendered the task even more difficult. The scientific field dedicated to this problem has been called “NLP”. In the 1980s and 1990s, NLP gained renewed interest due to technological advances that led to the development of more advanced AI systems [4]. Researchers designed a new generation of statistical AI models capable of learning from vast amounts of text data. These models were a significant improvement from previous rule-based systems and generated responses that sounded more natural [4]. The field of NLP underwent a significant transformation in the 2010s [5, 6]. Indeed, the widespread availability of internet text data and the development of deep learning methods changed the way scientists approached the problem of natural language understanding. Large-scale deep learning models such as recurrent neural networks and transformer models were trained on vast text datasets, enabling them to produce highly realistic human-like responses [6]. These models learned the nuances and structure of language from the input data, making them more effective than previous statistical models [6].

One of the most widely known and commonly used deep learning models is GPT, developed by OpenAI in 2018 [7]. The GPT model was trained on massive amounts of internet text data and was capable of generating responses that closely resembled human writing [7]. It quickly became popular for use in chatbots and other conversational AI applications. OpenAI subsequently developed even more sophisticated models, such as GPT-2 in 2019 and GPT-3 in 2020, which were capable of generating text that was almost indistinguishable from human writing and excelled in various language/ style tasks [8]. Despite the effectiveness of ChatGPT and other NLP technologies, they were relatively unknown to the scientific community for a while. However, in November 2022, OpenAI made multiple updates and improvements to ChatGPT, enhancing its ability to handle a wider range of queries and provide more accurate, relevant, and helpful responses to users [2]. The improvements included more training data, improved language understanding, an expanded knowledge base, and higher accuracy and precision [2]. The success of GPT and similar models has led to the development of conversational AI models by other companies and research organizations. For instance, Google’s Bidirectional Encoder Representations from Transformers (BERT) and Facebook’s RoBERTa models (a reimplementation of BERT with some modifications to the key hyperparameters and minor embedding tweaks) were trained on even larger text datasets and achieved state-of-the-art results in a range of NLP tasks [9, 10].

### Is NLP, including ChatGPT, really a source of concern?

There are several concerns about how ChatGPT may impact education, especially in academic writing. While ChatGPT is capable of generating essays on various topics, its scholarly writing still needs improvement, as evidenced by its performance when given an exam and a final project from a science denial class at George Washington University [11]. Nonetheless, the advent of AI in education may encourage instructors to rethink their teaching methodologies by providing assignments that demand critical thinking and problem-solving beyond the actual capabilities of AI that will evolve, probably in an endless race with humans. Another, more significant worry is the possibility of AI-generated content infiltrating scientific papers [2, 11, 12]. In a 2023 study, reviewers identified only 63% of the fake abstracts created by ChatGPT [12]. This raises pertinent issues concerning the authenticity and credibility of research publications, which is backed by an exclusively human based reviewer process as of now. The Science family of journals has always required authors to sign a license certifying that their work is original [11]. Interestingly, to ensure that this standard is met, the license and editorial policies have recently been updated to explicitly prohibit the use of AI-generated text, figures, images, or graphics in research publications. Moreover, AI programs cannot be considered authors of scientific papers. While AI tools can undoubtedly aid scientific research in many ways, it is crucial to recognize that they should function as a supplementary aid rather than a complete substitute for human creativity and ingenuity. Moreover, there are certain responsibilities that all authors of scientific research papers bear, which cannot be assumed by any computer or program.

### How can academic efficiency benefit from NLP, including ChatGPT?

ChatGPT offers numerous advantages that make it a valuable tool for academic research. One of its greatest strengths is its ability to process vast amounts of textual data in a short period, which can save researchers significant time and effort. In fact, ChatGPT and other NLP technologies have the potential to automate many tasks that were previously carried out manually. For instance, ChatGPT can be utilized to analyse academic papers by scanning them and extracting important details such as the author(s), publication date, and significant findings [11, 13]. This feature not only saves time but also enables researchers to avoid the tedious manual searching of papers. Another benefit of using ChatGPT in academic research is in the creation of summaries. Summarizing lengthy academic papers can be a time-consuming process, but ChatGPT can be trained to automatically generate them [12, 14, 15]. This feature guarantees an objective and unbiased summary, generated by a machine instead of a human (drawbacks and caution are discussed below). Furthermore, researchers can also use ChatGPT to generate research questions. By inputting a topic or research area into ChatGPT, researchers can obtain a list of potential research questions [2]. This feature can be particularly useful for new researchers or those seeking inspiration for a research project. However, it is important to use ChatGPT with caution and in conjunction with other research methods [12]. Researchers should carefully consider the scope of their research questions, be aware of the limitations of ChatGPT (see below), take care to attribute sources appropriately, exercise caution with sensitive or controversial topics, and remain informed about new developments in NLP.

### Will ChatGPT replace academic researchers?

ChatGPT is a robust LLM with various capabilities, yet it also possesses limitations that make it unsuitable for certain academic research types [2, 12]. The utilization of ChatGPT in research could result in the integration of false or biased information into papers, potentially leading to unintentional plagiarism and/or the misattribution of concepts. Moreover, researchers employing LLMs like ChatGPT run the risk of not adequately citing original sources or authors, leading to inadvertent misattribution of information [2]. Furthermore, the lack of transparency in the training sets and LLMs underlying ChatGPT and other conversational can cause biases and inaccuracies [2]. Importantly, the dearth of transparency and inability to access the internal workings of these models is contradictory to the current trend of transparency and open science. Additionally, concerns persist that conversational AIs can be influenced by the biases of their developers and training data, resulting in inaccuracies and incomplete knowledge. Van Dis et al. [2] demonstrated an instance of ChatGPT’s potential inaccuracies in their endeavour to summarize a systematic review on cognitive behavioural therapy for anxiety-related disorders. ChatGPT generated a response containing factual errors and misrepresentations, which could be attributed to the absence of relevant articles in its training set and/or the incapacity to differentiate between credible and less credible sources. To address these concerns, it is vital for researchers to maintain vigilance and integrate expert-driven fact-checking and verification processes into their work. Additionally, high-quality journals may opt to include human verification steps or technologies capable of identifying LLMs’ interference. The development and implementation of open-source AI technology present another means of addressing transparency and accuracy concerns [2]. Non-commercial organizations and academic collaborations have already started to create open-source LLMs that can promote innovation and reliability [2, 16]. Tech companies may also benefit from releasing relevant portions of their models and corpora as open source, encouraging greater community involvement and enabling the creation of more accurate and comprehensive results [2]. Similarly to how modern technology has not replaced engineers, computer scientists, or transportation companies, but rather improved their efficiency; academic researchers will not lose their importance with the advent of NLP technology. Instead, they will hopefully continue to grow, improve, and adapt. Hence, we firmly hold the view that NLP cannot substitute academic researchers because it could jeopardize research activities as a whole. ChatGPT has recently brought more light on one of the most pressing challenges faced by academic researchers, namely the threat of ‘fake science’. Scientists shall adapt to contemporary changes while continuing to excel as they have always done throughout history.

### What should be done in regard to NLP, including ChatGPT? A call for action

Primarily, we recommend that researchers, reviewers, editors, and publishers should try out ChatGPT for themselves. This will allow them to explore the capabilities of such a program and hopefully follow its development, keeping a close eye on any potential pitfalls or issues. Ultimately, we hope that they will act as guardians of honest science. In this regard, educators should discuss the use and ethics of this technology with undergraduate students. In the absence of any external guidelines so far, responsible group leaders and teachers should determine how to use ChatGPT with honesty, integrity, and transparency and establish some basic rules of engagement [12]. All contributors to research should be reminded that they would be held accountable for their work, whether it was generated with the support of ChatGPT/equivalent support, or not. Therefore, every author should take responsibility for thoroughly fact checking their text, results, data, code(s), and references. One additional critical issue to address is the implications for diversity and inequality in the research field. LLMs could be a double-edged sword [12]. Indeed, and importantly, LLMs could help level-off the playing field by removing language barriers and hopefully enabling more people to write high-quality text. However, it is likely that high-income countries and privileged researchers will quickly find ways to exploit LLMs in ways that accelerate their research and widen inequalities even more. Here, we urge the managers and developers to consider these global/language inequities and integrate in the development of future tools features that will provide equal chances to people independently of their language and access to technologies. Therefore, we propose that the U.S. NLM (https://www.nlm.nih.gov/) should create software similar to a plagiarism checker (“NLP pattern checker” could be suggested as a name) to help editors and publishers detect text generated by LLMs instead of researchers and/or reviewers. We foresee an epic battle between ChatGPT’s developers and whoever will be in charge of detecting non-human created text. In that regard, and as of now, ChatGPT’s raw output can be detected on careful inspection, particularly when the text contains more than a few paragraphs and the subject relates to scientific work. This is because LLMs produce patterns of words based on statistical associations in their training data and prompts, meaning that their output can appear generic or contain simple errors.

### Should ChatGPT be mentioned in the list of authors?

There are growing concerns regarding the ethical use of LLMs such as ChatGPT in academic research. There is a risk that researchers and students may rely too heavily on these models, resulting in unfair and unreliable work and/or even plagiarism [2]. To address these concerns, some scientific publishers, such as Springer *Nature*, have already established guidelines for ethically using LLMs. These guidelines advise against crediting LLMs as authors on research papers since, as mentioned above, AI tools/computers cannot take responsibility for the work. Researchers are also encouraged to document their use of LLMs in the methods or acknowledgements sections of their papers [17]. As an illustration of the latter [18], we asked ChatGPT to generate the abstract of the present manuscript (Box 1). Despite these guidelines, some papers have already credited LLMs as co-authors, such as an editorial in *Nurse Education in Practice* [19]. However, despite the honest behaviour of the authors to credit the help of ChatGPT, the journal’s editor-in-chief has further acknowledged this as an oversight from the journal editorial board and has promised to make the necessary corrections [20]. Another paper, published in *Oncoscience*, credited ChatGPT as a co-author of a perspective article [21]. The author of the paper, the chief executive officer of *Insilico Medicine*, strikingly stated that his company has published over 80 papers produced by generative AI tools [21]. While some argue that LLMs can produce higher quality work than earlier generations of AI tools, there are still concerns about their ethical use in research. Publishers such as Springer Nature have established guidelines to promote ethical and transparent use of LLMs while holding researchers accountable for their work [22]. To maintain transparency, it is essential to acknowledge the use of any NLP software, including simpler tools such as Grammarly and ProWritingAid, in the methodology or acknowledgement section of all manuscripts. This recommendation applies not only to ChatGPT and other NLP technologies but should also apply to publishers, editors, and reviewers who should encourage transparency in their own reviews and feedback to authors. Basic fairness would naturally result in acknowledging the contribution of any tool that aided in producing better research outcomes.

BOX 1 Verbatim version of the abstract created by ChatGPT based on the article manuscript final version (Date: March 5, 2023). Readers will identify ChatGPT actual limitations in constructing a well-structured abstract that covers all aspects of the article.

ChatGPT is a powerful tool for academic research with the ability to process vast amounts of textual data and automate tasks that were previously done manually. It can be used to scan academic papers and extract important details, generate objective and unbiased summaries, and create research questions. However, researchers must exercise caution when using ChatGPT as it can also integrate false or biased information into papers, potentially leading to unintentional plagiarism and misattribution of concepts. The lack of transparency in the training sets and underlying models can also cause biases and inaccuracies. To address these concerns, researchers should maintain vigilance, integrate expert-driven fact-checking and verification processes, and encourage the development and implementation of open-source AI technology. Educators should also discuss the use and ethics of this technology with undergraduate students, and all contributors to research should take responsibility for thoroughly fact-checking their work. Additionally, the implications for diversity and inequality in the research field should be considered, and the development of future tools should aim to provide equal chances to people regardless of their language and access to technologies. Finally, the proposal for the creation of a plagiarism checker similar to NLP pattern checker to help detect text generated by LLMs is suggested to help editors and publishers detect potential issues. Overall, ChatGPT has the potential to improve the efficiency of academic research, but researchers must use it responsibly and with caution to avoid any unintended consequences.

**Note from the authors:** While ChatGPT may prove to be beneficial to researchers; it is still a long way from being able to replace academics.

Furthermore, we believe that grey literature, including pre-prints, should not be considered as references for the time being. This is because it may contain significant bias due to being totally or partially generated by AI tools. We would like to emphasize that we are not opposed to the use of AI technology in academic writing, but it is important to note that manuscripts that have not undergone a robust peer review process may increase the risk of failing to identify AI-generated/supported articles. This means that academics who serve as reviewers should make the necessary efforts to follow the evolution of LLMs in order to reduce the likelihood that machines will imperceptibly replace humans in research.

### Is the use of NLP, including ChatGPT, by academic researchers any different in the sports science field?

We searched MEDLINE on March 4, 2023, using the combination of the following two terms: ChatGPT AND Sport. Only two editorials were found [23, 24]. The use of ChatGPT in academic research, including sports science, has both advantages and disadvantages that are not fundamentally different from other academic fields. One potential limitation is that ChatGPT was trained on a small academic database [25], which may not capture the full breadth and depth of knowledge in sports science. Furthermore, ChatGPT has been trained on a dataset that ends in September 2021 [25], while the academic sports science field is rapidly evolving, with new research emerging at an unprecedented pace. Within a year, evidence can quickly become outdated as new discoveries and advances are made. This emphasizes the importance of keeping up to speed with the latest research and constantly reassessing and updating scientific knowledge in the field, which increases the potential for bias in ChatGPT’s responses to sports science questions. Likewise, much of the online sports science information may be biased or based on anecdotal evidence rather than empirical research. This is because much of the content available on the internet is created by non-experts or individuals with a vested interest in promoting specific ideas or products. As a result, the quality and reliability of the available information may limit ChatGPT’s responses to sports science questions. Moreover, the popularization of sports science has resulted in an increase in the availability of biased or inaccurate information on the internet [26]. Athletes and fitness enthusiasts frequently share their experiences and advice, which may or may not be supported by empirical research and may be influenced by commercial interests. As a result, ChatGPT should prioritize peer-reviewed academic sources and be sceptical of non-academic sources in order to ensure the accuracy and reliability of its responses. This is especially important in sports science research, where inaccurate or biased data can have serious consequences for athletes’ health and performance.

The limitations of ChatGPT, however, are not unique to sports science research. In many fields, the internet is rife with biased or inaccurate information, and researchers must always be cautious when evaluating online sources. Furthermore, overreliance on ChatGPT-generated responses is a risk in any academic field. While ChatGPT can offer useful insights into research questions, it should not be used as the sole source of analysis. In conjunction with other research methods, researchers should always critically evaluate and interpret ChatGPT-generated responses. To illustrate our discussion with a practical example, the integral answer of ChatGPT to the question *“How can we use ChatGPT in sports?’*’ is reported in Box 2. We deliberately decided to keep Box 2 as is, even though at the time of our manuscript writing-up (February 2023), ChatGPT’s database was set up to September 2021 only. Therefore, statements as “*Providing realtime updates*”, should obviously considered with caution.

BOX 2 Verbatim’ answer of ChatGPT to the following request: “How can we use ChatGPT in sports?’’ (Access date, February 28^th^ 2023).

**Table:** 

**ChatGPT’s answer:**	

ChatGPT can be used in sports in several ways, such as:	

	ChatGPT can be used to:

*1. Providing real-time updates*	Provide real-time updates on live sports events, such as scores, highlights, and player statistics. This can help fans stay up-to-date on their favourite teams and players.

*2. Answering fan questions*	Answer fan questions about sports, such as rules, regulations, and historical facts. This can help fans better understand the sport and appreciate it more.

*3. Providing personalized training programs*	Provide personalized training programs for athletes based on their individual needs and goals. This can help athletes improve their performance and achieve their full potential.

*4. Conducting market research*	Conduct market research on sports fans, such as their preferences, opinions, and behaviours. This can help sports organizations better understand their audience and tailor their offerings to meet their needs

*5. Providing sports betting tips*	Provide sports betting tips and predictions based on historical data and statistical analysis. This can help bettors make more informed decisions and improve their chances of winning

### How easy is it to fool reviewers, editors, journals, and publishers with fake papers?

It is worth noting that in 2013, *The Guardian* newspaper/website published an article which detailed a sting operation conducted by the science journalist John Bohannon [27]. Over a period of 10 months, Bohannon submitted a fake scientific paper to 304 open access journals worldwide. The hoax paper had “fatal flaws” and used fabricated content, authors and universities. Surprisingly, and worryingly, the fabricated text was accepted by 157 journals, including those published by industry giants Sage, Elsevier, and Wolters Kluwer. The sting operation raised concerns about the quality control of open access journals and the “gold” open access model. The article suggested that the hoax reflected a weakness in peer review rather than a flaw in the gold open access model. Nowadays, we interpret that journal article differently. Indeed, with the excellent academic skills of NLP technology, less-skilled reviewers are less likely to detect or distinguish fake from true scientific articles. This will inevitably increase the threat of fake science.

It is now clear how easy it is to fool reviewers, editors, journals, and publishers with fake papers. Therefore, the three main messages of our article are as follows:

- NLP, including ChatGPT, should no longer be considered a threat to academics, as they have always existed, and their output continues to improve, just like scientific researchers’ output will do. Their existence will contribute to the improvement of science, but only if high-quality ethical standards are applied
- We call for highly skilled academics and pioneering leaders in big academic institutions to impose higher standards for academic writing, such as double author identification verification, or criteria for each author to submit in relevant journals. The same goes for peer reviewers; upon reviewers’ selection, editors may rely on their H-index or the number of their reviews to increase the standards, double institutional review board verification, and/or banning pre-prints or imposing more criteria for deposition before accepting pre prints (we believe that this is less likely to happen; therefore we call on the research community to avoid relying on pre-print manuscripts to back the research)
- It is essential to include a module for medical students, as well as for master’s and PhD students, on NLP technologies, ethics, and the best way to use them.

When asked to “*consider coming up with tools that will protect science and academics from the misuse of LLMs*”, the integral answer of ChatGPT is reported in Box 3. The authors call on the readers to judge whether ChatGPT did a satisfactory job for the assignment. Our personal opinion is that the text is really good.

BOX 3 Verbatim’ answer of ChatGPT to the following request: Please consider coming-up with tools that will protect science and academics from the misuse of large language models (LLMs) (access date, February 20^th^ 2023 – Importantly, this outcome is strictly independent from the content of the present manuscript. The authors would like to emphasize that we asked ChatGPT to answer this question after having completed our paper draft. We were surprised by some text sections that were extremely close to our manuscript, and ensure the readers that we did not take the below responses into account to bring any modification to our article).

**Table:** 

**ChatGPT’s answer:**

There are several steps that can be taken to protect science and academics from the potential misuse of LLMs like ChatGPT. Here are a few suggestions: *Educate researchers and academics*: It is crucial to educate researchers and academics about the capabilities and limitations of LLMs, as well as the potential risks associated with their use. This could involve providing training on best practices for using LLMs in research and emphasizing the importance of maintaining academic integrity. *Develop guidelines and standards*: The research community could develop guidelines and standards for using LLMs in academic research. This could include standards for verifying the authenticity of research outputs and guidelines for detecting and addressing potential misuse of LLMs. *Develop tools for detecting AI-generated content*: It is important to develop tools that can detect AI-generated content in scientific publications and research outputs. This could involve developing algorithms that can identify language patterns and other characteristics of AI-generated text. *Promote responsible use*: It is crucial to promote responsible use of LLMs in academic research. This could involve developing ethical guidelines for the use of LLMs in research and encouraging researchers to use LLMs in a way that supports human intelligence and critical thinking, rather than replacing it. *Engage with the broader community:* It is important to engage with the broader community, including policymakers and the public, to raise awareness about the potential risks and benefits of LLMs in academic research. This could involve developing educational materials and hosting public discussions to foster informed and responsible use of LLMs in research. Overall, protecting science and academics from the potential misuse of LLMs will require a collaborative effort from the research community, policymakers, and the broader public. By working together, we can develop the tools, guidelines, and standards needed to ensure that LLMs are used in a responsible and ethical manner, in support of scientific research and academic excellence.

## CONCLUSIONS

With the growth of AI technology, science, like many other fields, is not only greatly benefiting, but also currently facing challenges to its core principles, methods, norms and ethics. The priority should be to embrace the opportunities presented by this development and optimally manage any associated risks. It is believed that science can make the most of conversational AI while still maintaining the essential elements that make it such a rewarding and significant pursuit, such as curiosity, creativity, and exploration. Actually, the progress of AI technology may limit and obscure people’s/researchers’ contributions in the future. Indeed, AI chatbots may be able to generate research questions and hypotheses, develop methodology, create experiments’ research protocols, analyse and interpret data, and write manuscripts. Although we are still some way off from this scenario, there is no doubt that conversational AI technology will increasingly impact all stages of the scientific publishing process. Indeed, we believe that research institutions and academics will not be able to exclude the use of this technology. Therefore, this is a call for action for academics to act in the field of academic research. They shall focus on educating research students on the basic principles and ethical considerations involved in academic research. It is also important to emphasize the transparency of their work by acknowledging the use of any AI/LLMs at any stage of their research. Additionally, researchers should always crosscheck any information provided by these technologies with relevant sources. In doing so, researchers can ensure the ethical and transparent use of these tools in academic research. The race is now ongoing between humans and LLMs to come up with the best ways to limit the threats of such new AI-based tools while optimizing the benefits they will bring to humanity. Box 4 summarises the main messages of this review.

BOX 4 Main messages of this paper.

**Table:** 

***Message 1. Is NLP technology, including ChatGPT, really a new concept?*** The evolution of ChatGPT and similar products is closely linked to the development of AI and NLP. From the early days of rule-based systems to today’s cutting-edge deep learning models, the field has made tremendous strides in generating highly realistic human-like text and performing a wide range of language tasks.

***Message 2. Is NLP, including ChatGPT, really a source of concern?*** While ChatGPT’s capabilities offer exciting prospects for education and research, there are potential hazards associated with its usage that deserve full consideration. Maintaining the integrity of scientific research necessitates strict adherence to ethical and academic principles, with human intelligence and critical thinking at the forefront of the research process.

***Message 3. How can academic efficiency benefit from NLP, including ChatGPT?*** While ChatGPT has several benefits that make it a promising and effective tool for academic research, researchers must exercise caution and use it appropriately in conjunction with other research methods with cautious checks all along the way, to ensure trustful/reliable results.

***Message 4. Will ChatGPT replace academic researchers?*** While ChatGPT and other LLMs have numerous potential uses in academic research, they are not suitable for all types of research and pose accuracy and transparency threats/risks. Researchers employing these tools must maintain vigilance and integrate expert-driven fact-checking and verification processes into their work. Importantly, if some field of data science could be taken in charge by computers, most of the research experiments need humans to be ran. Thankfully, we do not expect humans to be completely replaced by computers yet.

***Message 5. What should be done in regard to NLP, including ChatGPT? A call for action*** We propose that the U.S. NLM creates a software similar to a plagiarism checker to help editors and publishers detect text generated by LLMs instead of researchers and/or reviewers.

***Message 6. Should ChatGPT be mentioned in the authors’ list?*** Current guidelines advice against crediting LLMs as authors on research papers since, as mentioned above, AI tools/computers cannot take responsibility for the work. However, if the authors use such tools, it would be fair mentioning it in the acknowledgements’ section of the manuscripts.

***Message 7. Is the use of NLP, including ChatGPT, by academic researcher any different in the sports science field?*** The advantages and disadvantages of using ChatGPT in academic research for sports science are not fundamentally different from the advantages and disadvantages of using ChatGPT in other academic fields. ChatGPT has the potential to shed light on important issues in sports science research, but its limitations must be considered and addressed. In conjunction with other research methods, researchers should prioritize the use of peer-reviewed academic sources and critically evaluate and interpret ChatGPT-generated responses.

***Message 8. How easy it is to fool reviewers, editors, journals, and publishers with fake papers?*** It is essential to include a module for medical students, as well as for master’s and PhD. students, on NLM technologies, ethics, and the best way to use them.

**AI**: Artificial Intelligence. **LLM**: Large Language Model. **NLM**: National Library of Medicine. **NLP**: Natural Language Processing.
